# The Pregnancy and Influenza Multinational Epidemiologic (PRIME) study: a prospective cohort study of the impact of influenza during pregnancy among women in middle-income countries

**DOI:** 10.1186/s12978-018-0600-x

**Published:** 2018-09-21

**Authors:** Fatimah S. Dawood, Danielle Hunt, Archana Patel, Wanitchaya Kittikraisak, Yeny Tinoco, Kunal Kurhe, Giselle Soto, Danielle Hombroek, Shikha Garg, Tawee Chotpitayasunondh, Oswaldo Gonzales, Savita Bhargav, Mark G. Thompson, Bajaree Chotpitayasunondh, Richard Florian, Amber Prakash, Sofia Arriola, Louis Macareo, Prabir Das, Santiago Cabrera, Sayda La Rosa, Eduardo Azziz-Baumgartner, for the Pregnancy and Influenza Multinational Epidemiologic (PRIME) Study Working Group*, Meredith Wesley, Pat Schifflet, Tana Brummer, William Campbell, Joshua Mott, Damon W. Ellison, Vaishali Kerdikar, Shailendra Mundhada, Madhavi Deshmukh

**Affiliations:** 1 Influenza Division, United States Centers for Disease Control and Prevention, 1600 Clifton Rd MS A-32, Atlanta, GA 30329 USA; 2 Abt Associates, Atlanta, GA USA; 3 Lata Medical Research Foundation, Nagpur, India; 4 Influenza Program, Thailand Ministry of Public Health – U.S. Centers for Disease Control and Prevention Collaboration, Nonthaburi, Thailand; 5 Naval Medical Research Unit No. 6, Bellavista, Peru; 6 Queen Sirikit National Institute of Child Health, Thailand Ministry of Public Health, Bangkok, Thailand; 7 Instituto Nacional Materno Perinatal, Lima, Peru; 8 Hospital Nacional Arzobispo Loayza, Lima, Peru; 9 Armed Forces Research Institute of Medical Sciences, Bangkok, Thailand; 10 Hospital Nacional Docente Madre Niño San Bartolomé, Lima, Peru

**Keywords:** Influenza, Respiratory infection, Pregnancy, Premature birth, Birth weight

## Abstract

**Background:**

The World Health Organization identifies pregnant women as at high-risk for severe influenza, but influenza vaccines are underutilized among pregnant women. Data on influenza burden during pregnancy are largely limited to high-income countries and data on the impact of influenza on birth and perinatal outcomes are scarce.

**Methods/design:**

This prospective, longitudinal cohort study of pregnant women in middle-income countries is designed to address three primary objectives: 1) to evaluate the effect of laboratory-confirmed influenza during pregnancy on pregnancy and perinatal outcomes; 2) to estimate the incidences of all-cause acute respiratory illness and laboratory-confirmed influenza during pregnancy; and 3) to examine the clinical spectrum of illness associated with influenza viruses. Through a multi-country network approach, three sites aim to enroll cohorts of 1500–3000 pregnant women just before local influenza seasons. Women aged ≥ 18 years with expected delivery dates ≥ 8 weeks after the start of the influenza season are eligible. Women are followed throughout pregnancy through twice weekly surveillance for influenza symptoms (≥ 1 of myalgia, cough, runny nose, sore throat, or difficulty breathing) and have mid-turbinate nasal swabs collected for influenza virus testing during illness episodes. Primary outcomes include relative risk of preterm birth and mean birth weight among term singleton infants of women with and without reverse transcription polymerase chain reaction-confirmed influenza during pregnancy. Gestational age is determined by ultrasound at < 28 weeks gestation and birth weight is measured by digital scales using standardized methods. Sites are primarily urban in Bangkok, Thailand; Lima, Peru; and Nagpur, India. All sites recruit from antenatal clinics at referral hospitals and conduct surveillance using telephone calls, messaging applications, or home visits. Nasal swabs are self-collected by participants in Thailand and by study staff in Peru and India. During the first year (2017), sites enrolled participants during March–May in Peru and May–July in India and Thailand; 4779 women were enrolled.

**Discussion:**

This study aims to generate evidence of the impact of influenza during pregnancy to inform decisions by Ministries of Health, healthcare providers, and pregnant women in middle-income countries about the value of influenza vaccination during pregnancy.

## Plain English summary

Studies show that pregnant women are at an increased risk for influenza-associated hospitalizations, but little is known about the incidence of influenza and influenza-associated complications during pregnancy. In addition, the few studies that have examined the effect of influenza during pregnancy on birth outcomes have mixed results with some studies finding an association between influenza and pregnancy loss, preterm birth, and poor fetal growth, while others find no association. There are no published studies from low- or middle-income countries of the effect of seasonal influenza during pregnancy on birth outcomes, even though pregnancies in these countries account for > 90% of births globally.

The Pregnancy and Influenza Multinational Epidemiologic Study is a large prospective study that enrolls pregnant women during multiple years in middle-income countries and follows these pregnant women through the end of their pregnancies to evaluate the effect of influenza during pregnancy on birth outcomes. The study is conducted at sites in India, Peru, and Thailand. Data are collected about participants’ medical histories, symptoms of respiratory illness during pregnancy, and birth outcomes including standardized measurement of gestational age and birth weight. The study also includes collection of nasal swabs from women who have respiratory illness during pregnancy to test for influenza virus infection. The study aims to generate data on the frequency and impact of influenza during pregnancy in middle-income countries to provide policy makers, healthcare personnel, and pregnant women with evidence to make decisions about the use of influenza vaccines during pregnancy.

## Background

Influenza viruses cause annual epidemics of respiratory infection globally. Pregnant women are thought to be at increased risk for morbidity and mortality from respiratory infections, including seasonal influenza, because of changes in anatomy and the immune and cardiovascular systems that accompany pregnancy [1, 2]. Data from high-income countries indicate that pregnant women are at increased risk of hospitalization because of influenza compared to non-pregnant women of childbearing age and the general population [3, 4]. Risks of influenza-associated hospitalization during pregnancy also appear to increase by trimester, with women in their third trimesters at greatest risk [3, 4].

Although studies show that pregnancy confers an increased risk for influenza-associated hospitalizations, few data are available about the incidence of influenza and influenza-associated complications during pregnancy. Influenza virus infection is hypothesized to increase the risk of preterm birth and fetal growth restriction through the ensuing inflammatory response and/or by triggering immune dysregulation that may activate pathways precipitating early labor and alter placental transfer to the fetus of nutrients or cytokines that influence metabolism [5–7]. However, studies on the effect of influenza during pregnancy on perinatal outcomes have mixed results [8, 9] with some studies finding an association between influenza and stillbirth, spontaneous abortion, preterm birth and fetal growth restriction [10–14] while others find no association [15]. There are no studies from low- or middle-income countries of the effect of laboratory-confirmed seasonal influenza during pregnancy on perinatal outcomes [8], despite the fact that pregnancies in these countries account for > 90% of births each year [16]. Data from low- or middle-income countries on the incidence of influenza virus infection during pregnancy are only available from the placebo or control arms of a few randomized controlled trials evaluating influenza vaccines during pregnancy [17–19].

Influenza vaccines are the most effective method of influenza prevention. Studies document that influenza vaccines are safe [20, 21] and effective [17–19, 22–24] at preventing influenza among pregnant women. Despite the recommendation by the Strategic Advisory Group of Experts (SAGE) on Immunization in 2012 to prioritize pregnant women for expansion of influenza vaccination programs [25], influenza vaccines remain underutilized or are not used at all among pregnant women in many countries [26]. Data about the incidence and impact of influenza virus infection during pregnancy could inform decisions about the potential value of influenza vaccines for pregnant women, particularly in low- and middle-income countries that will likely weight the introduction of influenza vaccines against other potential public health prevention programs. If findings demonstrate the impact of influenza virus infection during pregnancy on birth and perinatal outcomes, including increased infant morbidity and mortality, this would likely provide policy makers, healthcare personnel, and pregnant women with compelling evidence about the value of influenza vaccination [9].

The Pregnancy and Influenza Multinational Epidemiologic (PRIME) study is a prospective, longitudinal cohort study of pregnant women in three middle-income countries designed to address three primary objectives: 1) to evaluate the effect of laboratory-confirmed influenza virus infection during pregnancy on pregnancy and perinatal outcomes; 2) to estimate the incidences of all-cause acute respiratory illness, febrile acute respiratory illness, and laboratory-confirmed influenza virus infection during pregnancy; and 3) to examine the clinical spectrum of illness associated with influenza viruses, including duration and severity of illness.

## Methods/design

### Site characteristics

Sites in low- or middle-income countries were evaluated and selected based on prior experience enrolling pregnant women into research studies; ability to access local data on pregnancy and birth outcomes to guide sample size assumptions; ability to enroll at least 1000 pregnant women during a three month period as supported by previous study enrollment experiences and/or data on number of pregnancies or live-births in the study area; access to local reverse transcription polymerase chain reaction (RT-PCR) laboratory testing for influenza using U.S. Centers for Disease Control and Prevention (US CDC) protocols; ability to measure primary outcomes using standardized methods described below; access to at least three years of local influenza surveillance data to establish the typical timing of seasonal influenza epidemics; and ability to implement real-time electronic data collection in the field. Sites were also selected based on an anticipated low uptake of influenza vaccine since effective influenza vaccination would be expected to impact influenza virus infection incidence. The PRIME study network includes three sites: Thailand Ministry of Public Health – U.S. Centers for Disease Control and Prevention Collaboration, Queen Sirikit National Institute of Child Health, Armed Forces Research Institute of Medical Sciences, and two collaborating hospitals in Bangkok, Thailand; the U.S. Naval Medical Research Unit No. 6 and four collaborating hospitals in Lima, Peru; and Lata Medical Research Foundation and one collaborating hospital in Nagpur, India. Site characteristics are described in detail in Table 1.

**Table 1:** Site characteristics, the Pregnancy and Influenza Multinational Epidemiologic Study

	Bangkok, Thailand	Lima, Peru	Nagpur, India
Primary research institution(s)	Queen Sirikit National Institute of Child Health, Thailand Ministry of Public Health Thailand Ministry of Public Health—US Centers for Disease Control and Prevention Collaboration	U.S. Naval Medical Research Unit No. 6 (NAMRU-6)	Lata Medical Research Foundation
Study laboratory^a^	Armed Forces Research Institute of Medical Sciences	NAMRU-6 Virology and Emerging Infections Laboratory	Dhruv Pathology and Diagnostic Laboratory (year 1)
Source population	Pregnant women attending outpatient prenatal care visits at study hospitals	Pregnant women attending outpatient prenatal care visits at study hospitals	Pregnant women from urban Nagpur attending outpatient prenatal care visits at study hospitals
Study hospitals			
Number	2	4	1
Types	Tertiary/referral	Tertiary/referral	Secondary/referral
Institutions (deliveries/year)	Nopparat Hospital (6000) Rajavithi Hospital (6000-7000)	Peruvian Maternal and Perinatal Institute (22000) Arzobispo Loayza National Hospital (4000) Dos de Mayo National Hospital (4000) San Bartolome Hospital (7000)	Daga Memorial Government Women’s Hospital (14000-15000)
Start date of local influenza season^b^ (epidemiologic week)	28	18	26

^a^Study laboratories process and test respiratory specimens for influenza viruses by reverse transcription polymerase chain reaction (RT-PCR) using US Centers for Disease Control and Prevention protocols. In Thailand, the study laboratory also tests for respiratory syncytial viruses and human metapneumoviruses by RT-PCR

^b^For the purposes of recruitment and enrollment, the ‘influenza season’ is defined as the 16 week time period in which influenza epidemics are most likely to occur as predicted by local influenza virus surveillance data from previous years

### Design overview

The PRIME study follows women from enrollment during pregnancy through 6–8 weeks postpartum (Table 2). Site investigators recruit and enroll pregnant women into the study just prior to or at the beginning of the influenza season. At enrollment, participants complete an enrollment interview and are scheduled to undergo transabdominal ultrasound for pregnancy dating if they have not already had an ultrasound at < 28 weeks gestation with pregnancy dating results that can be verified by medical records.

**Table 2:** Data collection activities, the Pregnancy and Influenza Multinational Epidemiologic Study

Data collection activity	Enrollment	Pregnancy	Delivery/end of Pregnancy	Postpartum (6–8 weeks after end of pregnancy)	At any time point
Enrollment interview^a^	X				
Designation of proxy^b^	X				
Orientation to illness reporting activities^c^	X				
How to report ILS episodes					
Symptom diary card					
Digital thermometer use					
Self swabbing (Thailand only)					
Knowledge, attitudes and practices survey	X				
Ultrasound data collection form^d^	X				
Whole blood collection (serology sub-study participants)^e^	(X)		(X)		
Twice weekly surveillance contacts to identify ILS and delivery/end of pregnancy^f^		X			
		If ILS identified:			
Symptom screening log^g^		(X)			
Illness follow-up interview^h^		(X)			
Respiratory specimen collection^i^		(X)			
Delivery/end of pregnacy interview^j^			X		
Birth weight data collection form^k^			X		
Postpartum interview^l^				X	
Influenza vaccination verification^m^				(X)	
End of pregnancy/delivery chart abstraction^n^				X	
Acute hospitalization chart abstraction^o^					(X)
Death reporting form					(X)
Withdrawal form					(X)

ILS: Influenza like symptoms defined as new onset or sudden worsening of one or more of the following symptoms: myalgia, cough, runny nose or nasal congestion, sore throat, or difficulty breathing

Parentheses indicate activities that only occur for certain participants

a The enrollment interview collects information on socio-demographic characteristics, past medical and pregnancy history, and prenatal care for the current pregnancy

b During the enrollment process, each participant is asked to designate one or more proxies who may provide certain information about the participant’s health and pregnancy if she is not reachable by study staff. Study staff contact the participants’ proxy(ies) in the following situations: 1) if they are unable to reach the participants for twice weekly surveillance contacts after three attempts, 2) if they are unable to reach the participants for the end-of-pregnancy interview or post-partum interview, or 3) in the event of participant death reported by other sources (e.g. hospital staff)

c All participants receive a study orientation that emphasizes that the study aims to capture all potential respiratory illness episodes during participants’ pregnancies, accurately measure body temperature during illness episodes, and capture pregnancy outcomes such as infants’ birth weights as close to delivery as possible. They are given information about how to contact study staff with questions or notify them of an ILS episode, digital thermometers and symptom diary cards with instruction on using these items during ILS episodes. At the Bangkok site, instruction and demonstration on preforming self-collection of a mid-turbinate nasal swab (i.e., “self-swab”) and “swabbing kits” are also provided

d All participants have an ultrasound data collection form completed close to the time of enrollment. The form collects information on the parameters used for gestational age dating and the estimated gestational age in weeks and days

e Sites in Lima and Bangkok participate in a serology sub-study in which the sites aim to enroll 700 women per year to have whole blood collected at enrollment and within 7 days after the end of pregnancy to assess seroconversion to circulating strains during pregnancy

f Study staff contact participants twice weekly throughout their pregnancies to ascertain whether women have experienced ILS or end of pregnancy. Contacts are made by telephone call, messaging application or home visit

g If a participant reports an illness potentially meeting ILS criteria, staff conduct a symptom screening log to confirm illness symptoms and time since symptom onset

h Participants with confirmed ILS episodes are contacted starting seven days after illness onset (and every other day up through 13 days after illness onset) to determine when the ILS has ended. Once they report that their ILS has ended or it is 13 days after symptom onset, they complete an illness follow-up interview in person or by telephone that collects information on symptoms and subjective severity, duration of fever and height of highest measured temperature, missed work and/or absence from daily activities, receipt of medical care, and use of antiviral or antibiotic prescriptions

i Participants whose ILS episodes began within the past 7 days have a mid-turbinate nasal swab collected for influenza virus testing within 24 h of illness report. Participants at the Bangkok site self-collect their swabs

j Study staff administer a questionnaire within 7 days after delivery or end of pregnancy to collect information on prenatal course and delivery outcomes

k If participants deliver in a study hospital or clinic, infants are weighed on the day of delivery with study-approved digital scales. If participants deliver at a non-study hospital or at home, study staff attempt to visit the woman on the day of delivery (but no later than 48 h after delivery), when feasible, to measure the infant’s birth weight using study digital scales. In the minority of instances when a particpiant delivers at a non-study hospital and study staff cannot visit the woman, weights are abstracted from available medical records. All weights are recorded to the nearest gram

l The postpartum interview is conducted at 6–8 weeks after end of pregnancy and collects information on postpartum and neonatal course

m For participants who report receiving influenza vaccination, study staff attempt to verify vaccination status against medical records or vaccine registries

n Study staff abstract information from any available medical records on prenatal care, pregnancy course, and delivery/perinatal outcomes

o If a woman is hopitalized during her pregnancy for reasons other than delivery, study staff attempt to abstract information on the acute hospitalization from available medical records

Throughout cohort participation, study staff instruct and remind participants to contact staff if they experience influenza-like symptoms (ILS). ILS is defined as new onset or sudden worsening of one or more of the following symptoms: myalgia, cough, runny nose or nasal congestion, sore throat, or difficulty breathing; the surveillance case definition was chosen to be sensitive for detection of influenza and include both respiratory and constitutional symptoms. Active surveillance for ILS is also conducted twice weekly via one or more site-specific contact methods, including telephone calls, mobile telephone text messaging, or home visits by study staff. Participants with illness episodes meeting the ILS case definition complete acute illness and illness follow-up interviews. If symptom onset occurred within the last 7 days, mid-turbinate nasal swabs are collected by trained study staff (Peru and India) or participant self-collection (Thailand) [27–29]. Local CDC-approved laboratories test nasal swab specimens for influenza viruses by real-time reverse transcription polymerase chain reaction (rRT-PCR) using CDC protocols.

Study staff contact participants within 7 days of the end of the pregnancy (i.e. delivery, spontaneous abortion, elective abortion, or stillbirth) to complete a survey about their pregnancy course and perinatal outcomes. If women deliver in a study hospital, study staff or hospital personnel trained on study procedures weigh infants on the day of delivery using study-approved digital scales. If infants are born at a non-study hospital or outside the hospital setting, study staff attempt to visit the woman on the day of delivery (but no later than 48 h after delivery) when feasible to measure the infant’s birth weight using study-approved digital scales. If medical records of the end of pregnancy are available, study staff abstract data for select pregnancy and perinatal outcomes. If women report receipt of influenza vaccination during their pregnancy, study staff also verify influenza vaccine receipt against available records. At 6–8 weeks postpartum, women also complete a survey on postpartum and neonatal course. Additional site specific methods are described in detail in Table 3.

**Table 3:** Site-specific methods, the Pregnancy and Influenza Multinational Epidemiologic Study

	Bangkok, Thailand	Lima, Peru	Nagpur, India
Recruitment and enrollment			
Enrollment period, year 1 (epidemiologic weeks)	20–32	11–22	19–30
Additional eligibility requirements	Ability to speak and understand Thai	None	Resident of urban Nagpur, Access to mobile phone
Recruitment setting	Antenatal care clinic	Antenatal care clinc, Preventive care medicine clinic	Antenatal care clinic
Method of identifying potentially eligible women	Review of medical record and antenatal clinic cards	Staff maintain list of all women seen in the clinics each day	Staff maintain list of all women seen in the clinics each day
Method of sampling women for recruitment	None, attempt to approach all women	Sample every 3rd woman during high patient volume periods, otherwise, attempt to approach all women	None, attempt to approach all women
Gestational age dating by ultrasound			
Personnel primarily responsible for performing ultrasound	Hospital ultrasonographers trained on gestational age dating SOP	Hospital ultrasonographers trained on gestational age dating SOP	Private ultrasonographers hired for study and trained on gestational age dating SOP
Surveillance for ILS			
Primary method of contact with participants	Telephone calls	Telephone calls	Telephone calls, Home visits
Additional methods used by participants to report illness	Telephone calls to study nurses	Telephone calls or text messaging application to study staff	Telephone hotline to study staff Customized electronic application
Method of nasal swab collection	Participant self-collection	Study staff collection	Study staff collection
End of pregnancy or delivery			
Methods of identifying end of pregnancy or delivery	Surveillance contacts, Medical records flagged so hospital staff recognize study participants at delivery and can communicate with study team	Surveillance contacts, Review of labor room records	Surveillance contacts, Telephone hotline for participants and family to call Medical records flagged so hospital staff recognize study participants at delivery and can communicate with study team Review of labor room records
Primary location of deliveries	Hospital	Hospital	Hospital
Birthweights, personnel primarily responsible for collection	Delivery nurses trained on birthweight collection SOP	Delivery nurses trained on birthweight collection SOP	Delivery nurse midwives and study staff trained on birthweight collection SOP
Chart abstraction data sources	Delivery hospitalization records Antenatal clinic records	Delivery hospitalization records Antenatal clinic records National prenatal care card	Delivery hospitalization records Antenatal clinic records
Influenza vaccine verification sources	Hospital records	National prenatal care card Hospital records	Hospital records Antenatal clinic records Immunization cards
Additional substudy activities			
Serology specimen collection for serology substudy	Yes	Yes	No
Testing nasal swabs for other respiratory viruses	Yes (hMPV, RSV)	No	No

*SOP* Standard operating procedures, *hMPV* human metapneumovirus, *RSV* respiratory syncytial virus

### Eligibility

Women are eligible to participate if they are pregnant as confirmed by urine pregnancy test and/or abdominal ultrasound; are 18 years of age or older; have an estimated date of delivery (EDD) based on last menstrual period (LMP) or ultrasound that allows for 8 weeks of observation during the influenza season if the pregnancy continues to 40 weeks gestation; have had an ultrasound at < 28 weeks gestation to establish EDD at a facility where medical records are available for verification or are currently at < 28 weeks gestation based on reported EDD or LMP and willing to undergo a transabdominal ultrasound for pregnancy dating as part of the study; plan to remain in the study area for the duration of pregnancy and plan to deliver at study hospitals; and are willing and able to be contacted twice weekly from enrollment through the end of pregnancy for study surveillance purposes. At the Bangkok site, women must also be abe to speak and understand Thai (Table 3).

### Enrollment

Study staff enroll participants at each site over a period of up to 14 weeks extending from 10 weeks before the anticipated start of the influenza season through the first four weeks of influenza season. For the purposes of enrollment, the influenza season is defined as the 16 week period in which influenza epidemics are most likely to occur as predicted by local influenza virus surveillance data from previous years [30–32]. The goal is to enroll the cohort just prior to or at the beginning of the influenza season so each cohort member will be under observation for the full period that she is pregnant during the influenza season.

All sites recruit women from local antenatal care clinics (ANCs). Study staff approach potentially eligible women for eligibility screening while women are waiting to be seen for ANC appointments or while awaiting ultrasound appointments. Women provide written informed consent to study participation prior to study screening.

### Study proxies

During the enrollment process, participants are offered the option to designate one or more proxies who may provide certain information about the participants’ health and pregnancy if they are not reachable by study staff. Study staff contact the participants’ proxy(ies) in the following situations: 1) if they are unable to reach participants for twice weekly surveillance contacts after three attempts, 2) if they are unable to reach participants for the end of pregnancy interview or post-partum interview, or 3) in the event of participant death reported by other sources (e.g. hospital staff). The primary purpose of contacting the study proxy during missed surveillance or interview contacts is to ascertain whether the participant’s illness has progressed in severity to the point that she is unable to respond to study staff and/or whether the participant has gone into labor, delivered, or otherwise experienced an end to her pregnancy.

### Study procedures and data collection

At enrollment, study staff instruct participants that they aim to capture all potential respiratory illness episodes during participants’ pregnancies, accurately measure body temperature during illness episodes, and document pregnancy outcomes such as infants’ birth weights as close to delivery as possible. Staff provide participants with 1) information about how to contact staff with questions or notify them of an ILS episode; 2) digital thermometers and symptom diary cards with instruction on use during ILS episodes; and 3) at the Bangkok site, “swabbing kits” and instruction and demonstration about performing self-collection of a mid-turbinate nasal swab (i.e., “self-swab”). Women then complete an enrollment interview about socio-demographic characteristics, past medical and pregnancy history, and prenatal care for the current pregnancy. Women also complete a questionnaire about knowledge, attitudes and practices related to influenza illness and vaccination.

Study staff then contact participants twice weekly throughout their pregnancies to ascertain whether women have experienced an ILS episode or end of pregnancy. Staff remind women with ILS episodes to complete a symptom diary card, measure their oral temperature daily prior to taking antipyretics, and document the impact of illness on their daily activities. For women with ILS, study staff contact women every other day starting seven days after illness onset up through 13 days after illness onset to determine when their ILS episode has ended. Once women report that their ILS episode has ended or at the end of 13 days from symptom onset, women complete an illness follow-up interview in person or by telephone; information is collected about symptoms and subjective severity, duration of fever and height of highest measured temperature, missed work and/or absence from daily activities, receipt of medical care, and use of antiviral and antibiotic medications.

Within 7 days after the end of pregnancy, women complete an interview that collects information about pregnancy complications, prenatal care and health behaviors since enrollment, end of pregnancy complications and outcomes, and updates on influenza vaccination status. Staff contact women once more at 6–8 weeks postpartum to complete a brief interview on postpartum medical visits or hospitalizations by the mother or infant and infant health status since delivery.

When medical records are available, study staff complete a chart abstraction form after the end of pregnancy to collect information on prenatal care, pregnancy course, delivery/perinatal outcomes, and any hospitalizations for acute medical illnesses that occur during the pregnancy. Medical records are also used to verify receipt of influenza vaccine for women who report receiving influenza vaccine during their pregnancies. In the event that a participant or her infant dies, study staff complete a death data collection form to compile information about the location and nature of the death from verbal report and official death records, when available. Deaths are also reported to local Institutional Review Boards according to local reporting requirements.

### Outcomes

Primary outcomes of the study comparing women with versus without RT-PCR-confirmed influenza virus infection during pregnancy include: 1) the relative risk of preterm birth (birth at < 37 weeks gestation); 2) difference in mean gestational age at delivery or end of pregnancy; and 3) difference in mean birth weight among all infants and among term singleton infants. Additional primary outcomes include episodes of RT-PCR-confirmed influenza per 10,000 pregnant women months of observation, frequency of illness signs and symptoms, and duration of measured fever ≥ 38.0 degrees Celsius among women with RT-PCR-confirmed influenza (Table 4).

**Table 4:** Pregnancy and Influenza Multinational Epidemiologic Study Primary and Secondary Objectives and Outcomes

Objectives	Outcomes^a^
Primary Objectives	
Evaluate the effects of laboratory-confirmed influenza virus infection during pregnancy on pregnancy and perinatal outcomes.	• Relative risk of preterm birth (< 37 weeks gestation based on ultrasound performed at < 28 weeks gestation) among women with versus those without real- time reverse transcription polymerase chain reaction (RT-PCR)-confirmed influenza virus infection during pregnancy (P) • Difference in mean gestational age at delivery or end of pregnancy among women with versus those without RT-PCR-confirmed influenza virus infection during pregnancy (P) • Difference in mean birth weight among all infants and among term singleton infants born to women with versus those without RT-PCR-confirmed influenza virus infection during pregnancy (P)
Estimate the incidences of any acute respiratory illness (ARI), febrile ARI, and laboratory-confirmed influenza during pregnancy.	• Episodes per 10,000 pregnant women months of observation of: o RT-PCR-confirmed influenza (P) o serologically-confirmed influenza (P) o ARI (S) o febrile ARI (S) o any ARI-associated hospitalization (S) o RT-PCR-confirmed influenza-associated hospitalization (S)
Examine the clinical spectrum of illness due to influenza virus infection among pregnant women including duration and severity of illness.	• Frequency of illness signs and symptoms and duration of measured fever > 38.0 C among pregnant women with RT-PCR-confirmed influenza (P)
Secondary Objectives	
Evaluate the effects of ARI and febrile ARI during pregnancy on pregnancy and perinatal outcomes among women in middle-income countries.	• Difference in mean gestational age at delivery or end of pregnancy among women with versus without any ARI or febrile ARI (as separate exposures) during pregnancy (S) • Difference in mean birth weight among all infants and among term infants born to women with versus without any ARI or febrile ARI (as separate exposures) during pregnancy (S) • Relative risk among women or infants of women with versus without any ARI or febrile ARI (as separate exposures) during pregnancy of o spontaneous abortion (< 22 weeks gestation) (S) o stillbirth (> = 22 weeks gestation) (S) o maternal death during pregnancy or within 28 days of end of pregnancy (S) o early neonatal death (at 0–6 days of life) (S) o neonatal death (at 0–27 days of life) (S)
Examine knowledge, attitudes, and practices (KAP) related to influenza and influenza vaccination among pregnant women in middle-income countries.	• Frequencies of KAP related to influenza virus infection during pregnancy and its impact on the mother and fetus • Frequencies of KAP related to influenza vaccination during pregnancy, including safety and effectiveness of the vaccine for the mother and fetus • Frequencies of trusted sources of information about influenza and influenza vaccination during pregnancy • Association between KAP related to influenza and influenza vaccination and reported influenza vaccine receipt during the current pregnancy (at sites where influenza vaccines are available)
Estimate the effectiveness of inactivated influenza vaccine to prevent laboratory-confirmed influenza among pregnant women at sites where influenza vaccine is used in pregnant women.	• Relative risk of influenza virus infection among women who received influenza vaccine during pregnancy compared to those who did not receive vaccine
Compare the accuracy of last menstrual period (LMP) versus ultrasound at < 28 weeks gestation for pregnancy dating in the subset of women who recall their LMP.	• Mean difference in days between gestational age estimated from LMP compared to ultrasound at < 28 weeks gestation using ultrasound as the gold standard.

^a^Primary outcomes and seconday outcomes identified in the protocol are denoted by (P) and (S), respectively. Primary outcomes are those for which the study aims to have adequate statistical power to evaluate if sample size goals are met

### Gestational age and birth weight measurement

Since prematurity and birth weight are primary study outcomes, sites follow standard operating procedures to ensure accurate and uniform pregnancy dating and measurement of birth weight. Ultrasound performed at < 28 weeks gestation is used as the primary means of dating participants’ pregnancies. For participants that had a vaginal or trans-abdominal ultrasound at < 28 weeks gestation for pregnancy dating as part of routine clinical care, study staff abstract from the medical record the method of dating, estimated gestational age and EDD based on the ultrasound reading. For participants who have not already had an ultrasound for pregnancy dating or for whom ultrasound records are not available, trained ultrasonographers hired by the study perform a transabdominal ultrasound according to study standard operating procedures. Because the accuracy of gestational age dating by ultrasound decreases with increasing gestational age [33–35], ultrasounds done as part of the study are done as soon after enrollment as possible. Ultrasounds performed for study purposes focus on gestational age estimation and do not report the sex of the fetus(es) or congenital anomalies on data collection forms, to hospital staff, or to the participant or her family.

At study hospitals or clinics, investigators assess and approve scales in the labor and delivery area for acceptability for study purposes prior to study initiation. Scales must be digital, designed to measure weight with accuracy to the nearest 10 g, and be calibrated daily. Study staff and collaborating hospital staff are trained prior to study initiation and periodically throughout the study period on weight measurement procedures which include removing all clothing from the infant down to no clothing or a thin shirt and performing at least two birth weight measurements consecutively. Study staff measure a third weight if there is a difference of more than 20 g between the first two measurements.

### Sample size

The PRIME study aims to enroll 13,200 pregnant women over at least two influenza seasons. The estimated sample size is based on achieving adequate power to estimate relative risk of preterm birth among women with versus without RT-PCR-confirmed influenza during pregnancy. According to WHO estimates, the average prevalence of preterm births ranges from 9% in high-income countries to 12% in low-income countries [36, 37]. Therefore, we assumed a preterm birth prevalence of at least 10% among pregnant women without influenza virus infection in this study. Assuming an influenza attack rate among the cohort of 3–5% based on experience with other influenza studies and 10% cohort attrition, a sample size of 13,200 pregnant women would provide adequate statistical power to detect a relative risk of preterm birth of 1.3 to 1.4 among women with influenza virus infection during pregnancy compared to those without. Using the same assumptions about influenza attack rate and attrition, the same cohort size would also provide adequate power to detect a mean difference in birth weight of 100 g assuming a mean birth weight among women without influenza virus infection during pregnancy of 3000 g with a standard deviation of 500 g.

### Substudies

The Bangkok and Lima sites conduct a substudy to estimate the incidence of serologically-confirmed influenza virus infection and estimate the proportion of serologically-confirmed influenza virus infections that are not identified by RT-PCR as part of ILS surveillance among pregnant women. Each site enrolls 700 women from the main cohort into the substudy each year. Women who received the current season’s influenza vaccine preceding cohort enrollment are not eligible for the serology substudy. Participants in the substudy have whole blood collected at enrollment (i.e. pre-influenza season) and again within 7 days of the end of pregnancy. Blood specimens will be tested at a single central laboratory for antibodies to the predominant circulating strains and relevant historical strains of influenza at each site by hemagglutination inhibition (HI) assay using US CDC-approved protocols; specimens may also be tested by microneutralization (MN) assays.

The Bangkok site is also conducting sub-studies to estimate the incidences of respiratory syncytial virus (RSV) and human metapneumovirus (hMPV) infection during pregnancy and evaluate their effects on pregnancy and perinatal outcomes. Nasal swab specimens collected at the Bangkok site will be tested by RT-PCR for RSV and hMPV using US CDC protocols. For a subset of ILS episodes, the Bangkok site will also compare specimen quality and the number of viruses detected by participant-collection of nasal swabs versus staff-collection of nasal swabs for the detection of influenza viruses, RSV and hMPV [28].

### Laboratory methods

Local laboratories test nasal swab specimens by real time RT-PCR assays to ascertain infection with influenza A or B viruses and identify influenza A subtype (A/H1N1 and A/H3N2) and influenza B lineage (B/Yamagata and B/Victoria). Testing is completed using US CDC protocols and with primers, probes, and reagents provided by US CDC. Local study laboratories completed and passed either WHO or US CDC-approved proficiency panels for influenza RT-PCR testing within one year prior to starting testing for the PRIME study.

### Data management

Each site maintains study databases on site, including a separate database with patient identifying information and contact information, which is kept securely with respect to privacy, cybersecurity, and patient confidentiality. Each participant is assigned a unique identification number and all associated data is stored in the common study database using REDCap (Research Electronic Data Capture), which is a browser-based metadata-driven software system (Vanderbilt University, Nashville, TN). Enrollment, end of pregnancy, and post-partum interview data are collected in real-time using internet-enabled personal computers or mobile devices (tablets or smart phones). ILS surveillance data, medical chart and death record abstractions, and vaccination verification data are entered into REDCap regularly throughout data collection. Laboratory results are uploaded to REDCap routinely during periods of testing. REDCap data entry fields provide quality assurance through the use of logic and range checks and automated skip patterns. Additional routine quality checks of the data, such as checks for out-of-range values and missing data, are performed on a weekly basis centrally by the data coordinating center (Abt Associates Inc.)

At the Nagpur, India site, study staff use an android application installed on sim card enabled tablets to contact participants twice weekly. The application categorizes participants into city zones based on their residential address. Each study staff is allocated a zone and a list of participants from that zone that she needs to follow up for ILS. The application synchronizes with the enrolment data of the participants from REDCap to provide staff with a list of women that are due for a home visit, telephone call or respiratory specimen collection. The application also allows study staff to call participants’ phones directly from the tablet device and records responses in real time.

## Discussion

The PRIME Study is a multi-site collaboration that aims to generate data on the impact of influenza virus infection during pregnancy among women in middle-income countries to inform decisions about the value of influenza vaccination of pregnant women in these settings. Assessing the impact of influenza virus infection during pregnancy on adverse pregnancy outcomes requires enrollment of a large number of women because of the rarity of outcomes. It also requires a prospective design to avoid biases inherent in retrospective recall of infection and adverse outcomes and rigorous measurement of primary outcomes (e.g. birth weight and gestational age) that are vulnerable to measurement error during standard clinical practice. The PRIME Study seeks to meet these requirements through a collaborative network approach using a standardized protocol, data collection tools, and standard operating procedures. The study also uses centrally-designed trainings and frequent site monitoring to ensure adeherence to the the study protocol and procedures. The PRIME study will also generate multi-site and multi-year estimates of influenza virus infection incidence during pregnancy using standardized methods that allow comparison of estimates and a broad case definition to capture the full spectrum of illness from non-medically attended illness episodes to hospitalizations and deaths. These data will provide critical inputs needed to model burden and cost of influenza during pregnancy.

Enrolling a large number of pregnant women over a relatively short period of time and ensuring consistent and close follow-up for many months through the postpartum period is challenging. During 2017, the first year of PRIME study enrollment, the network successfully approached and screened 8034 women and fully enrolled 4779 women. A common challenge across sites was that women who experienced adverse pregnancy outcomes such as miscarriage or stillbirth were often less willing to remain in the study and provide information about the adverse outcomes. To overcome this challenge, sites spent substantial time training study staff on developing a strong rapport with participants, responding to adverse events with sympathy and information about why study participation might help physicians learn to decrease adverse outcomes for other women, and strategies to obtain key information on adverse outcomes from other sources (e.g. family members, the medical record) or from the participant at a later time point. Sites also found that obtaining birth weights on infants who were born with critical illness was difficult. Through strong collaborative partnerships with hospital administration and clinicians, study staff were able to find ways to collect at least one birth weight according to the study SOP in many such cases without delaying standard of care for critically ill infants. In addition, the Thailand site faced challenges meeting enrollment targets during the first year of the study because study hospitals serve immigrants who may not be eligible for the study because they are not fluent in Thai. Lastly, all study sites worked among populations in which women often returned to a family home in another location to give birth to their infants. Because collection of birth weights and other pregnancy outcome data was a key focus of the PRIME study, women were only eligible to participate in the study if they indicated that they planned to stay in the study area for delivery. Nevertheless, sites still found that a small proportion of women left the study area for delivery. In those cases, study staff either attempted to visit the place of delivery to collect outcome information themselves or obtain medical records from which outcomes could be verified. Study teams have also developed additional strategies to limit enrollment to women who will give birth in the study area during subsequent years of the study.

Major strengths of the PRIME study include its use of a standard protocol and rigorous outcome measurement and enrollment of a large sample of pregnant women that would be difficult to do at a single site. The PRIME study also demonstrates the feasibility of implementing real-time data collection, standard operating procedures for birth weight and gestational age measurement including ultrasonography in resource-limited settings, and novel methods of influenza surveillance such as participant-collected respiratory specimens and use of local text and tracking applications. An important limitation of the PRIME study is that it was not designed to estimate the incidence of severe influenza-associated outcomes such as hospitalization and death which are rare and would require a much larger sample size to assess. However, the PRIME study does collect data on these outcomes and will provide estimates of their prevalence.

## Conclusion

The PRIME study is poised to provide estimates of the incidence of influenza virus infection from diverse sites in three middle-income countries during multiple influenza seasons. The study also aims to enroll a large enough sample of pregnant women to assess the impact of influenza virus infection on clinically important pregnancy and perinatal outcomes, although influenza circulation patterns will influence whether the network achieves adequate statistical power to addres this study objective.
